# Moving Forward with Prisms: Sensory-Motor Adaptation Improves Gait Initiation in Parkinson’s Disease

**DOI:** 10.3389/fneur.2012.00132

**Published:** 2012-09-28

**Authors:** Janet H. Bultitude, Robert D. Rafal, Corinne Tinker

**Affiliations:** ^1^Centre for Functional Magnetic Resonance Imaging of the Brain, University of OxfordOxford, UK; ^2^INSERM Unit 864 Space and ActionBron, France; ^3^Wolfson Centre for Clinical and Cognitive Neuroscience, School of Psychology, Bangor UniversityBangor, UK

**Keywords:** prism adaptation, Parkinson’s disease, gait, rehabilitation

## Abstract

It is postulated that the decreased walking speed; small, shuffling steps; and “freezing” shown by patients with Parkinson’s disease could stem from an inability to tilt the body forward enough to provide sufficient forward propulsion. In two repeated-measures studies we examined whether adaptation to upward-shifting prisms, resulting in a downward after-effect, could improve gait initiation in healthy participants and patients with Parkinson’s disease. Faster forward stepping followed a brief (5 min) exposure period for patients, and a longer (20 min) exposure period for age-matched controls. Backward stepping was unchanged, and adaptation to downward-shifting prisms with control participants showed no effect on forward or backward stepping. These results suggest that adaptation of arm proprioception in the vertical plane may generalize to anterior-posterior postural control, presenting new possibilities for the treatment of gait disturbance in basal ganglia disorders.

## Introduction

J. Purdon Martin described human locomotion as “controlled falling,” in which the forward and downward shift in the center of gravity (COG) is arrested by the action of the frontally positioned leg (Martin and Hurwitz, 1962; Martin, 1963). He argued that some features of gait in basal ganglia disorders such as Parkinson’s disease stem from an inability to control the COG of the body, and that impaired step initiation and occurrence of freezing reflect an inability to tilt the body forward enough to provide sufficient forward propulsion. Martin observed that inducing a forward shift in COG, for example by asking a patient to carry a chair while walking, restored normal locomotion (Martin and Hurwitz, 1962).

Decreased forward movement of the COG in Parkinson’s disease has since been examined more formally. At the beginning of normal locomotion, the COG is displaced forward relative to the center of pressure (COP), and this is thought to be essential for gait initiation (Jian et al., 1993). This displacement is smaller for patients with Parkinson’s disease than age-matched controls (Jian et al., 1993; Martin et al., 2002; Hass et al., 2005), and the magnitude of the displacement is negatively correlated with symptom severity (Hass et al., 2005). The ability to shift the body forward over the feet – to set the “falling” in motion – may play a key role in determining the extent to which a patient’s walking is impaired.

The present research was prompted by evidence that postural imbalance in hemiplegic patients can be alleviated by sensory-motor adaptation to sideways prismatic shifts in the visual field (Tilikete et al., 2001). Patients with hemiplegia following unilateral brain damage – especially to the right cerebral hemisphere – often lean toward the ipsilesional side (De Oliveira et al., 2008). Tilikete et al. (2001) reported that adaptation to rightward prismatic shifts in the visual field reduced postural imbalance in such patients, as demonstrated by a more central COP following treatment. During prism adaptation participants reach to targets that are viewed through prismatic lenses that bend the light before it reaches the eyes, shifting the visual image to one side. Since their movements are programmed based on shifted visual information, participants initially point to one side of the target. With successive trials, however, pointing accuracy is re-established as visual, proprioceptive, and motor reference frames are realigned to shift pointing movements in the opposite direction of the visual distortion. Once the prisms are removed, this realignment is observed as pointing errors in the opposite direction to the prismatic shift: the adaptation after-effect.

For the present research we reasoned that adaptation to upward-shifting prisms using a similar procedure to that described above would induce downward pointing errors and forward postural shifts. This may in turn assist patients with Parkinson’s disease to achieve greater degrees of forward propulsion, reducing problems with gait initiation. Therefore, we examined the effect of vertical prism adaptation on gait initiation in both healthy individuals and patients with Parkinson’s disease. We adapted the prism adaptation protocols from the first studies to demonstrate changes in pen-and-paper test of spatial orienting. We first examined the effects of 20 min of adaptation to upward- or downward-shifting prisms on forward and backward stepping in healthy participants (Experiment 1), choosing this time period based on Michel’s et al. (2003a) first demonstration that adaptation to rightward-shifting prisms altered line bisection in healthy participants. Reaction times for forward stepping were significantly reduced after adaptation to upward-shifting prisms only, and reaction times for backward stepping were unchanged by adaptation to either upward- or downward-shifting prisms. Following on from this positive result, we examined the effect of 5 min of adaptation to upward-shifting prisms on stepping performance of 16 patients with Parkinson’s disease and 16 age- and sex-matched controls (Experiment 2). We selected this shorter adaptation period based on previous work demonstrating reduced neglect symptoms (Rossetti et al., 1998) and postural instability (Tilikete et al., 2001) in stroke patients who had adapted to rightward-shifting prisms for 5 min. We predicted that adaptation to upward-shifting prisms would reduce reaction times for forward stepping.

## Materials and Methods

### Participants

Participants for Experiment 1 were 20 older neurologically healthy individuals (6 males, mean age = 62, SEM = 1.1). Participants for Experiment 2 were 16 patients suffering from idiopathic Parkinson’s disease without dyskinesia (10 males, mean age = 66, SEM = 1.8) and 16 age- and sex-matched controls (mean age = 63, SEM = 2.9). All research procedures, including the obtainment of informed consent, complied with the Declaration of Helsinki and were approved by the ethical review committee of the School of Psychology at Bangor University.

The inclusion criteria for patients were (1) a diagnosis of idiopathic Parkinson’s Disease by a specialist in a movement disorder clinic; (2) age 18+. Exclusion criteria were (1) a history of neurological disease other than Parkinson’s Disease; (2) complete inability to walk; (3) impairment to walking for reasons other than Parkinson’s Disease; (4) impaired use of their right arm for reasons other than Parkinson’s Disease and to a degree of severity that would prevent the patient from undergoing prism adaptation; (5) a designation as legally blind. On the basis of these criteria, one patient was excluded from the study due to a co-morbid diagnosis of Multiple Sclerosis. The remaining 16 patients were formally assessed on the motor component of the Unified Parkinson’s Disease Rating Scale (UPDRS; mean score = 15/108, SEM = 2.0; see Table 1 for full clinical details). Medication details were obtained by means of self-report from the patients.

**Table 1:** **Clinical details of the patients with Parkinson’s disease who participated in Experiment 2**.

Patient	Sex	Age	Years since diagnosis	Medication (mg/day)	Minutes since last medication	UPDRS (motor component) scores				mTGUAG (secs)	Other
						Posture item (/4)	Gait item (/4)	Kinesia item (/4)	Total (/108)	
PD1	F	69	5	Pramipexole 1.08	200	2	2.5	2.5	27	31	S
PD2	M	64	12	l-DOPA 750, Carbidopa 75	330	1	2	2	24	22	F, S
PD3	M	59	1	Rasagiline 1, Ropinirole 9	180	0.5	0.5	0	6.5	15	
PD4	F	70	4	Pramipexole 2.2, l-DOPA 750, Carbidopa 75	345	1.5	2	2	19.5		
PD5	M	67	5	Pramipexole 2.1	260	2	2	2	15	21	
PD6	M	62	6	Pramipexole 3.1, l-DOPA 500, Carbidopa 50	105	2	1.5	1	14.5	16	
PD7	M	61	16	Pramipexole 4.5, Entacapone 200, l-DOPA/benserazide 875, Entacapone 1400	345 (first 2) and 105 (second 2)	1	0.5	1	19.5	13	
PD8	M	55	3	Pramipexole 2.82	150	0	0	0	3	12	
PD9	M	61	4	l-DOPA 400, Carbidopa 100	300	1.5	0.5	1	16	13	
PD10	F	59	17	Ropinirole l24	150	2.5	1.5	1.5	17	15	F, S
PD11	M	63	2	Ropinirole 9	180	0.5	0	0.5	10	14	
PD12	F	71	2	l-DOPA 750, Carbidopa 75	240	0	0	0.5	9.5	12	
PD13	F	71	5	Ropinirole 24, l-DOPA 200, Carbidopa25	120	1.5	0.5	1	16.5	17	S
PD14	M	81	10	l-DOPA 500, Carbidopa 100	120	1	0.5	1	21	27	F, S
PD15	F	68	1	l-DOPA 800, Carbidopa 200	150	1	1	2	30	20	F, S
PD16	M	72	5	Pramipexole 1.08, l-DOPA 100, Carbidopa 25	180	0	0	0	3	12	

*UPDRS, Unified Parkinson’s Disease Rating Scale; mTGUAG, A modified Timed Get-Up-And-Go test: patients rose from a chair without using their arms, walked for 4 m, turned around, walked back to the chair and sat down again. Normative data (*N* = 9, mean age = 65.2, SD = 4.8): CI^0.95^ = [11.8, 14.18]; F, patient reported occurrences of freezing; S, patient used a walking stick*.

### Stimuli and procedure

#### Stepping task

Participants in both experiments performed the same stepping task before and after prism adaptation. Participants stepped forward and backward in response to auditory cues while holding a 128 cm lever that was attached to a microswitch at floor level. The lever was held with the right hand with the arm held in a constant position with respect to the participant’s body such that the upper arm was in line with the torso, the elbow was at a right angle, and the forearm was parallel to the floor. The fixed positioning of the arm relative to the body was strictly monitored by the experimenter throughout the duration of the test. At the beginning of each trial participants stood in a natural stance with their feet adjacent to each other behind a line 28 cm behind the lever. The starting position was to the left of the lever location such that the right arm was parallel to the mid-sagittal plane. Stimuli were presented and reaction times recorded using E-prime experimental software. Each trial was triggered by the experimenter and began with a 500 ms tone. After a pause of 2000 ms a 750 ms pre-recorded vocal instruction of either “forward” or “backward” was played. Upon hearing the sound participants were required to step with their right foot in the indicated direction as quickly as possible. This caused an angular displacement of the lever, which triggered the microswitch once the deviation was larger than 9°. The reaction time was recorded as the time between the vocal instruction and the activation of the microswitch. Participants immediately returned to the starting position for the beginning of the next trial.

Thirty forward and thirty backward stepping trials were performed in pseudorandom order in each pre- and post-adaptation blocks. To discourage de-adaptation in the post-test phase, participants performed the gait initiation tests (both pre- and post-adaptation) blindfolded with an experimenter standing nearby to steady them if they lost their balance. Patients were given the option of performing the task without a blindfold if they felt too unsafe doing it blindfolded but none felt this was necessary. Training was performed before the experiment to ensure that patients fully transferred their weight with each step and maintained their arm in the correct position throughout the task.

#### Prism adaptation

A vertical prism adaptation task was devised based on standard lateral prism adaptation procedures used in previous studies (Rossetti et al., 1998; Tilikete et al., 2001; Berberovic and Mattingley, 2003; Michel et al., 2003a). A 90 cm wide × 35 cm high × 70 cm deep prism adaptation box was constructed based on that described by Berberovic and Mattingley (2003). The box was open at the top and two opposite ends. A vertical panel was fitted inside the box parallel with the participant’s torso and was used to provide vertical targets for the adaptation phase and also to allow the experimenter to measure the participant’s pointing errors. The position of this panel was adjustable to accommodate different arm lengths.

Participants were seated at one open end of the box with their chin resting on the top edge. To gain a baseline measure of vertical pointing in an open-loop condition the participants were first asked to close their eyes and point directly in front of their chin using the index finger of their right hand. This was devised as an approximate vertical equivalent of the standard “subjective straight ahead” measure of spatial realignment in the horizontal plane (e.g., Michel et al., 2003a), in which participants are asked to point straight ahead of their mid-sagittal plane. The experimenter measured the vertical displacement of their finger from the top of the box with the aid of the vertical panel. Pointing error was measured to the nearest 0.5° with a positive value indicating an upwards error and a negative value indicated a downwards error. Participants were asked to return their hand to resting directly in front of their torso between each pointing movement. Ten pointing measures were taken immediately before prism adaptation, immediately after prism adaptation, and after the completion of the post-adaptation stepping task (the “pre-,” “post-,” and “late-adaptation” open-loop pointing sessions, respectively).

For prism adaptation, the vertical panel was placed in front of the participant at arm’s length, upon which three targets were drawn at heights of 13.6 cm, 19.8 cm, and 24.9 cm from the bottom of the box (i.e., the upper-most target was approximately 10 cm below the participant’s chin). With their chin resting on the edge of the box, participants pointed with the index finger of their right hand to each of these targets in a pre-defined pattern (upper-middle-lower-middle) while wearing goggles that contained Risley biprisms set to induce a 15° shift in the visual image. In Experiment 1 half of the healthy participants adapted to upward-shifting prisms and half to downward-shifting prisms for a period of 20 min. In Experiment 2 all of the participants (both patients and controls) adapted to upward-shifting prisms for a period of 5 min. To help maintain constant pointing speed, participants pointed in time with a metronome set to 0.5 Hz. Participants returned their hand to rest in front of their torso between each pointing movement, and rested their arm as required. When the adaptation phase was completed the participants were asked to close their eyes and the experimenter removed the prism goggles.

## Results

Pointing errors and reaction time data for both experiments were evaluated using *a priori* paired *t*-tests with Bonferroni-corrected alpha levels.

### Experiment 1: Adaptation to upward- and downward-shifting prisms in healthy participants

#### Adaptation after-effect

**Figure 1:**
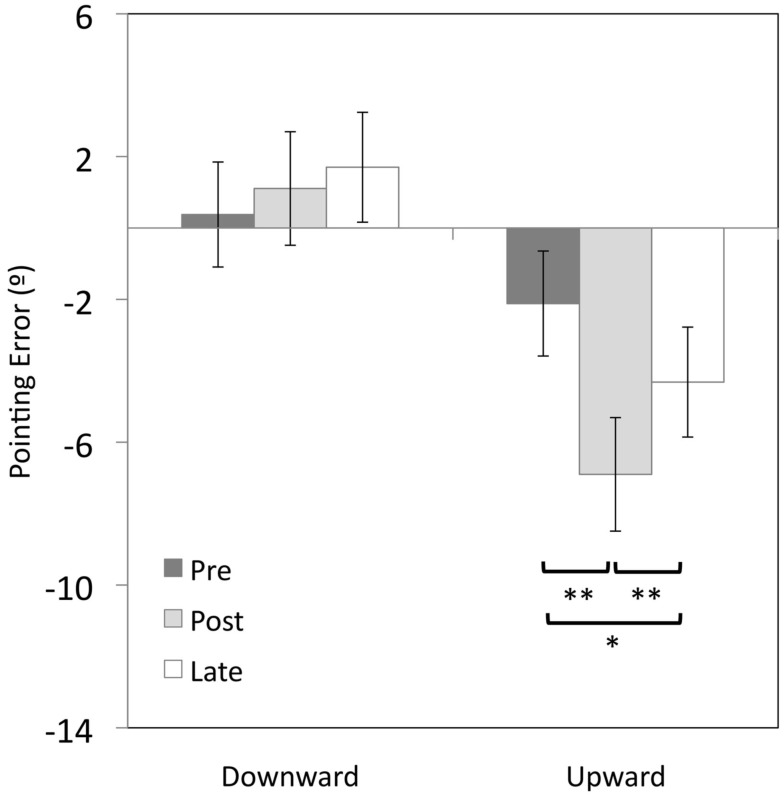
**Vertical errors (°) for open-loop pointing movements made by healthy participants in Experiment 1 immediately before (pre-test) and after prism adaptation (post-test), and after the completion of the post-adaptation stepping task (late-test)**. Positive values indicate upward errors, negative values indicate downward errors. *p < 0.05, **ps < 0.01, error bars represent ± 1 SEM.

Adaptation to upward-shifting prisms resulted in a significant 4.7° downward after-effect compared to baseline pointing (*t*(9) = 5.6, *p* < 0.001), with a significant 2.2° downward shift still evident at the late-test (*t*(9) = 3.3, *p* < 0.01; see Figure 1). However, there was no significant change in pointing errors following adaptation to downward-shifting prisms (*p*s > 0.05).

#### Stepping times

**Figure 2:**
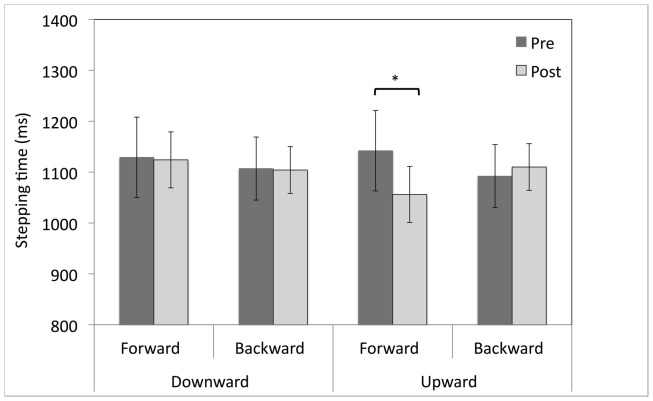
**Pre- and post-adaptation reaction times (ms) for forward and backward stepping for healthy participants after 20 min of adaptation to upward- or downward-shifting prisms**. *p < 0.05, error bars represent ± 1 SEM.

*T*-test comparisons of pre- vs. post-adaptation stepping initiation latency revealed that forward stepping was 86 ms faster after adaptation to upward-shifting prisms (*M* = 1142, SEM = 91 vs. *M* = 1056, SEM = 76; *t*(9) = 2.9, *p* < 0.05; see Figure 2). There was no significant change in forward stepping times following adaptation to downward-shifting prisms (*M* = 1129, SEM = 64 vs. *M* = 1124, SEM = 19; *t*(9) = 0.112, *p* = 0.914). Backward stepping times were unchanged by adaptation to either upward- or downward-shifting prisms (*p*s > 0.05).

### Experiment 2: Adaptation to upward-shifting prisms in patients with Parkinson’s disease

#### Adaptation after-effect

**Figure 3:**
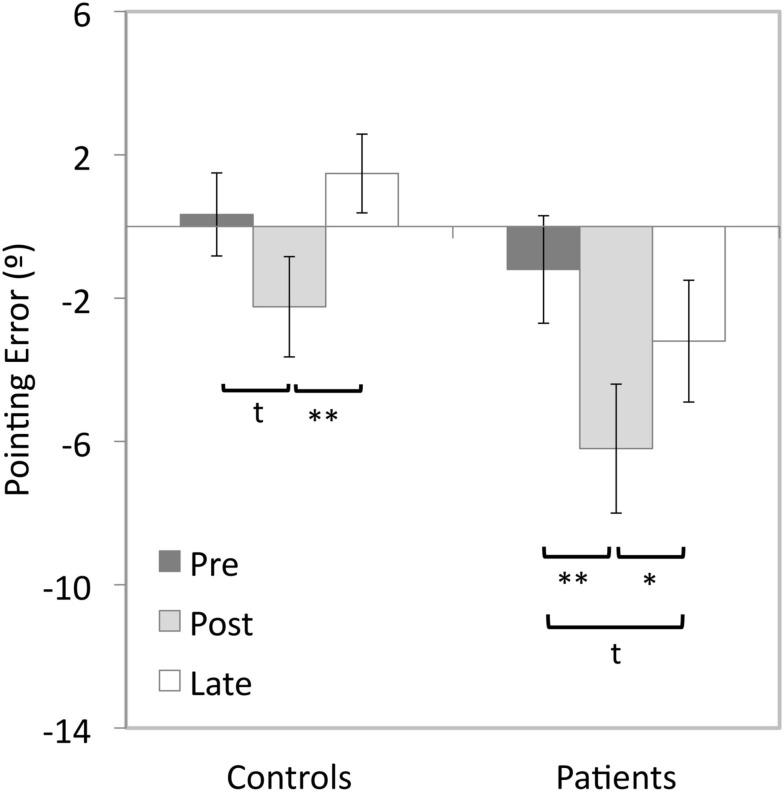
**Vertical errors (°) for open-loop pointing movements made after 5 min of adaptation to upward-shifting prisms by patients with Parkinson’s disease and control participants for the pre- post- and late-tests in Experiment 2**. Positive values indicate upward errors, negative values indicate downward errors. *p < 0.05, **ps < 0.01, *t* = trend, error bars represent ± 1SEM.

In patients, adaptation to upward-shifting prisms resulted in a significant 4.9° downward after-effect compared to baseline pointing (*t*(15) = 5.83, *p* < 0.001), with a 1.9° downwards trend in the pre- vs. late-adaptation comparison (*t*(15) = 2.41, *p* = 0.029, Bonferroni-corrected alpha level = 0.017; see Figure 3). In the control participants, a 2.6° downward shift in pointing between the pre- and post-adaptation tests approached significance (*t*(15) = 2.66, *p* = 0.018). However, there was no significant difference between pre- and late-adaptation pointing errors (*t*(15) = 1.5, *p* = 0.144).

#### Stepping times

An omnibus ANOVA of Group (PD, control) × Session (pre, post) × Direction (forward, backward) revealed a trend for a Group × Session interaction [*F*(1,30) = 3.97, *p* = 0.06], and a trend for a Session × Direction interaction [*F*(1, 30) = 3.36, *p* = 0.08]. Although the three-way interaction of Group × Session × Direction was not significant [*F*(1,30) = 2.55, *p* = 0.12], separate Group (PD, control) × Session (pre, post) ANOVAs were performed on the forward and backward stepping times on an *a priori* basis. There was a significant Group × Session interaction for forward stepping [*F*(1,30) = 5.67, *p* < 0.05], but not for backward stepping (*p* = 0.89).

**Figure 4:**
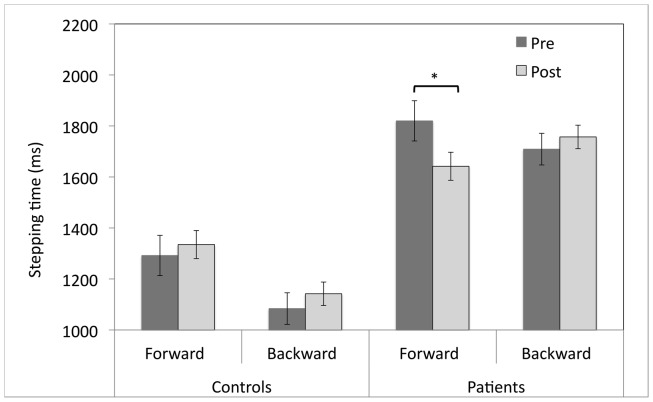
**Pre- and post-adaptation reaction times (ms) for forward and backward stepping for patients with Parkinson’s disease and control participants who adapted to upward-shifting prisms**. *p < 0.05, error bars represent ± 1 SEM.

*T*-test comparisons of pre- vs. post-adaptation stepping initiation latencies revealed that patients stepped forward 178 ms faster after adaptation to upward-shifting prisms (*M* = 1821, SEM = 215 vs. *M* = 1642, SEM = 171; *t*(15) = 2.2, *p* < 0.05; see Figure 4). However, there was no change in the forward stepping times for healthy participants (*M* = 1292, SEM = 93 vs. *M* = 1335, SEM = 79; *t*(15) = 0.92, *p* = 0.37). There were no changes in backward stepping times for either patient or control participants (*p*s > 0.05).

As there was a high degree of variation in the severity of symptoms in the patient sample group, a follow-up analysis was performed to determine if the above results varied with the degree of gait impairment. Patients were divided into two groups of equal size (*N* = 8) based on their performance on the mTGUAG test (“more impaired” group: mTGUAG ≥ 15 s, mean age = 69, SEM = 2.22; mean UPDRS motor score = 21.5/108, SEM = 1.8); “less impaired” group: mTGUAT < 15 s, mean age = 63, SEM = 2; mean UPDRS motor score = 10.3/108, SEM = 2.1). One patient who did not perform the mTGUAG test was place in the more impaired group based on her performance on the UPDRS gait item (2/4) and overall score (19.5/108). An ANOVA of patient performance with Level of Gait Impairment (more impaired vs. less impaired) as a between-groups factor and Session (pre, post) and Direction (forward, backward) revealed a main effect of Level of Gait Impairment [*F*(1, 14) = 14.1, *p* < 0.005], with significantly faster stepping times for the less impaired group (*M* = 2253, SEM = 196.5) than the more impaired group (*M* = 2253, SEM = 196.5). However, there were no significant interactions of Level of Gait Impairment with Session or Direction (*ps* > 0.05).

## Discussion

Our results provide a preliminary, proof-of-concept that adaptation to upward-shifting prisms may improve gait initiation in patients with Parkinson’s disease. Reaction times for forward stepping were significantly reduced following adaptation to upward-shifting prisms. There was no change in stepping times following adaptation to downward-shifting prisms, and neither adaptation to upward- nor downward-shifting prisms altered reaction times for backward stepping. In healthy older participants the effect required a longer period of adaptation: age- and sex-matched controls who adapted for 5 min showed no significant change in forward stepping times (Experiment 2), but older participants who adapted for 20 min had a significant reduction in forward stepping times similar to that observed in the patients (Experiment 1).

In Experiment 1, adaptation to downward-shifting prisms not only resulted in no change to stepping initiation times, but also failed to influence pointing after-effects. Changes in open-loop pointing error is a robust consequence of adaptation to lateral prismatic shifts in the visual field, therefore the absence of these low-level sensory-motor changes in the present study warrants some consideration. Although adaptation to laterally displacing prisms has been extensively studied in both healthy participants and clinical populations, little previous research has examined adaptation to vertical prismatic shifts. Martin et al. (2001) asked healthy participants to throw clay balls at visual targets viewed through downward-shifting prisms. The magnitudes of the throwing errors during initial prism exposure and immediately after the removal of the prisms were not significantly different to those found in a study by the same researchers using a similar procedure with leftward-shifting prisms (*p* > 0.05; Martin et al., 1996). This suggests it is unlikely that the fundamental mechanisms of horizontal and vertical adaptation are different. Instead, we suggest a much more practical explanation for the absence of upward after-effects in the downward-shifting prism group in our study. The long duration of the adaptation session in Experiment 1 (20 min) may have resulted in fatigue in the adapting arm and a subsequent downward drift in the arm position during pointing without visual guidance that opposed any upward after-effect in arm proprioception. Similar fatigue may also mean that the after-effect measures for the upward-shifting prism group are artificially inflated. Indeed, this explanation is supported by the fact that after-effects for control participants adapting to upward-shifting prisms were larger following 20 compared to 5 min of adaptation (4.7° vs. 2.6°). This possible confound could be prevented in future work by using another means of measuring the adaptation after-effect, such as a “visual straight ahead” measure in which participants are asked to indicate when a moving point of light is positioned directly in front of them (Redding and Wallace, 1997).

Present data do not enable us to determine whether the absence of any changes in stepping initiation following adaptation to downward-shifting prisms in Experiment 1 was because sensory-motor adaptation for that direction of visual shift does not generalize to posture and gait initiation, or because the sensory-motor realignment process normally undergone during prism adaptation was itself not successful. Nonetheless, this does not detract from our critical finding: that adaptation to upward-shifting prisms reduced stepping times for forward stepping in patients with Parkinson’s disease and healthy participants.

Our results are consistent with previous findings that initiation of gait in patients with Parkinson’s disease can be facilitated by simple changes in initial standing posture – specifically in the stance position of the initial swing arm (Dalton et al., 2011). We note, however, that arm stance *per se* would not explain the current results since the positions of both arms were kept stable throughout both the pre- and post-adaptation gait initiation task. Rather, we posit that a forward shift in body posture against the direction of the prismatic displacement – in keeping with the findings of Tilikete et al. (2001) – could have led to the observed change in gait initiation times.

The precise mechanism for this is uncertain. Prism adaptation of pointing movements was long held to have limited generalization to other types of movement. For example, adaptation of arm movements transferred to the ipsilateral leg (Savin and Morton, 2008), but not to walking trajectory (Morton and Bastian, 2004). Since the emergence of prism adaptation treatment for hemispatial neglect, however, broad-spread effects of prism adaptation on “higher-level” cognitive functions have been demonstrated (Michel et al., 2003a; Pisella et al., 2006). In contrast to pointing or walking after-effects, the extent of these changes do not seem to be related to the magnitude of the prismatic shift, and are asymmetrical in that they follow adaptation to only one direction of lateral visual shift (rightward-shifting prisms for stroke patients, and leftward-shifting prisms for healthy controls). These asymmetries suggest that the cognitive effects of prism adaptation involve the perturbation of right-hemisphere functions, such as spatial attention.

Tilikete and colleagues (Tilikete et al., 2001; Michel et al., 2003b) suggested that the effects of adaptation to laterally shifting prisms on postural stability was due to the updating of high-level representations of the body and space, rather than a straight-forward sensory-motor realignment. Like spatial attention and hemispatial neglect, the cognitive representation of the body has been linked to the right posterior parietal lobe (Pérennou et al., 2000; Pérennou, 2006). The changes in body posture following prism adaptation in the previous studies and our experiments may stem from perturbation of this internal model of the body structure.

In our current study, 20 min of adaptation was required to alter stepping initiation in healthy participants, whereas 5 min was sufficient to change performance in patients. This pattern is in keeping with existing literature that examined changes in spatial orienting following adaptation to laterally shifting prisms: for example, although Tilikete et al. (2001) demonstrated postural changes in stroke patients after only 5 min of prism adaptation, Michel et al. (2003b) used a 20 min adaptation period to demonstrate effects in healthy participants. Neglect patients who have undergone a brief period of adaptation in the order of 50 pointing movements cancel more targets on the left side of the page, and are more leftward in their estimation of the center of horizontal lines (Rossetti et al., 1998). These effects can last for up to one day after a single treatment session. Attentional changes in healthy individuals who have undergone prism adaptation are of a smaller magnitude than those demonstrated by patients (Michel et al., 2003a). Furthermore, although the longevity of the attentional effects in healthy individuals has not been formally tested, they are assumed to be short-lived and to be related to the duration of the visuo-motor after-effect. For this reason, studies involving healthy participants use longer adaptation periods. We have previously speculated that a longer exposure period is required for healthy participants because the normal equilibrium or “default” state of the intact brain is not as readily perturbed by prism adaptation as the altered neural environment of patients (Bultitude and Woods, 2010). It is possible that the repeat of this pattern in the present study is because the mechanisms through which adaptation to vertical prismatic shifts altered stepping may be similar to those by which adaptation to horizontal prismatic shifts alter spatial orienting.

Disturbances of gait in Parkinson’s disease, such as hesitation of gait initiation and freezing, may particularly contribute to a reduced sense of independence and increased disability – in part due to their intermittent and unpredictable nature (Smania et al., 2011). Indeed, Moore et al. (2007) found a negative correlation between the incidence of freezing and patients’ quality of life, suggesting that challenges to gait are of particular concern in the treatment of the disorder. In our results, the absence of any changes to backward stepping times following prism adaptation suggests that the observed effects are unlikely to stem from a general change in motor preparedness, but are specific to the forward direction of locomotion. This leads us to carefully consider that the protocol applied in the present study may not be helpful for all types of gait disturbance demonstrated by patients with Parkinson’s disease. For example, an intervention that shifts the COG forward could worsen symptoms of festination in susceptible patients.

It is frequently reported that symptoms of gait initiation impairment in Parkinson’s disease such as freezing and reduced stride length can be alleviated by the introduction of simple visual and auditory cues such as transverse lines drawn on the floor, or music with a rhythmic marching beat (Azulay et al., 1999; Jiang and Norman, 2006; Nieuwboer et al., 2009). The results of the present study suggest that prism adaptation may provide an equally simple means of reducing such gait impairment through *offline* treatment. A potential benefit of such an offline treatment is that it would generalize to a variety of situations, including those in which floor markings are unavailable, or listening to music is impractical. A sham-controlled trial employing direct measures of gait and posture will be critical for demonstrating the efficacy of such offline prism adaptation treatment.

Neither we, nor the patients who participated in the study, observed any noticeable improvements in gait following the prism adaptation session. This is perhaps not surprising: although improvements in hemispatial neglect have frequently been reported following single sessions of prism adaptation, the standard regime used for clinical rehabilitation involves once- or twice-daily treatment sessions for a total of 2 weeks (Frassinetti et al., 2002; Angeli et al., 2004; Serino et al., 2006, 2009). Stroke patients who have undergone such a treatment program show amelioration of symptoms compared to control groups that are sustained for up to 6 months post-treatment (Serino et al., 2006, 2007, 2009). A critical direction for future research would be to examine whether a similar regime may provide sustained benefits for patients with Parkinson’s disease, improving locomotion without the need for further daily use of prisms.

## Conflict of Interest Statement

The authors declare that the research was conducted in the absence of any commercial or financial relationships that could be construed as a potential conflict of interest.
